# Selective Cytotoxicity of Portuguese Propolis Ethyl Acetate Fraction towards Renal Cancer Cells

**DOI:** 10.3390/molecules27134001

**Published:** 2022-06-22

**Authors:** Ana Sofia Freitas, Marta Costa, Olívia Pontes, Veronique Seidel, Fernanda Proença, Susana M. Cardoso, Rui Oliveira, Fátima Baltazar, Cristina Almeida-Aguiar

**Affiliations:** 1 Centre for the Research and Technology of Agro-Environmental and Biological Sciences (CITAB), Department of Biology, University of Minho, 4710-057 Braga, Portugal; anasofiapfreitas@gmail.com; 2 Department of Biology, School of Sciences, University of Minho, Campus de Gualtar, 4710-057 Braga, Portugal; ruipso@bio.uminho.pt; 3 Centre of Molecular and Environmental Biology (CBMA), Department of Biology, University of Minho, 4710-057 Braga, Portugal; 4 Life and Health Sciences Research Institute (ICVS), School of Medicine, University of Minho, Campus of Gualtar, 4710-057 Braga, Portugal; martafcosta@med.uminho.pt (M.C.); oliviaepontes@gmail.com (O.P.); 5 ICVS/3B’s-PT Government Associate Laboratory, 4710-057 Braga/806-909 Guimarães, Portugal; 6 Natural Products Research Laboratory, Strathclyde Institute of Pharmacy and Biomedical Sciences, University of Strathclyde, Glasgow G4 0RE, UK; veronique.seidel@strath.ac.uk; 7 Department of Chemistry, University of Minho, Campus of Gualtar, 4710-057 Braga, Portugal; fproenca@quimica.uminho.pt; 8 LAQV-REQUIMTE, Department of Chemistry, University of Aveiro, 3810-193 Aveiro, Portugal; susanacardoso@ua.pt

**Keywords:** propolis, fractionation, phenolic compounds, pectolinarigenin, cytotoxic activity, renal cell carcinoma

## Abstract

Renal cell carcinoma is the most lethal cancer of the urological system due to late diagnosis and treatment resistance. Propolis, a beehive product, is a valuable natural source of compounds with bioactivities and may be a beneficial addition to current anticancer treatments. A Portuguese propolis sample, its fractions (*n*-hexane, ethyl acetate, *n*-butanol and water) and three subfractions (**P1**–**P3**), were tested for their toxicity on A498, 786-O and Caki-2 renal cell carcinoma cell lines and the non-neoplastic HK2 kidney cells. The ethyl acetate fraction showed the strongest toxicity against A498 (IC_50_ = 0.162 µg mL^−1^) and 786-O (IC_50_ = 0.271 µg mL^−1^) cells. With similar toxicity against 786-O, **P1** (IC_50_ = 3.8 µg mL^−1^) and **P3** (IC_50_ = 3.1 µg mL^−1^) exhibited greater effect when combined (IC_50_ = 2.5 µg mL^−1^). Results support the potential of propolis and its constituents as promising coadjuvants in renal cell carcinoma treatment.

## 1. Introduction

According to the GLOBOCAN 2020 database, despite not being one of the most prevalent cancers, renal cell carcinoma (RCC) is the most aggressive and lethal cancer of the urological system. By 2040, its incidence and mortality are expected to increase by approximately 54 and 68%, respectively [1]. Metastatic RCC is very frequently identified, mainly due to late diagnosis, and its aggressiveness requires a combination of surgery and systemic treatment, which is associated with important adverse effects [2]. Therefore, alternative treatment approaches are needed to provide more effective treatments and/or to reduce the toxicity associated with systemic treatment.

The demand for natural products has been increasing due to their importance in human health and their potential as new drug leads [3]. Propolis is a natural product produced by bees as a building material and defensive substance in the hive [4]. The chemical composition of propolis is complex and comprises mainly resinous and balsamic material collected from branches, flowers, pollen, buds and exudates of trees mixed with substances resulting from bees’ metabolism [4]. Propolis is a very popular natural remedy, widely recognised as an important source of compounds that exhibit a range of biological properties, including anticancer activity [5], being commonly available as a food supplement with diverse beneficial effects mostly due to its high flavonoid content [6]. Several compounds have been identified in different propolis samples, with phenolics being the most important. The flavonoids pinocembrin, galangin and chrysin and phenolic acids such as caffeic acid, ferulic acid and cinnamic acid are the most common phenolic compounds in propolis from temperate zones [7,8]. Propolis from Gerês has been studied by our research group since 2011, being characterized by the prevalence of the same compounds such as chrysin, caffeic acid isoprenyl ester (CAIE) and pinocembrin, found in abundance, and other compounds such as pinobanksin and phenolic acids derivatives, found in lower amounts [9].

The cytotoxic effect of propolis has been extensively studied against diverse types of cancer cell lines, including breast [10,11], prostate [12], colon [13], melanoma and colorectal [14,15] cancer cells, as well as on other cell lines [5,16]. However, few studies have explored the potential of Portuguese propolis for the treatment of different types of cancer [14,16,17]. Propolis activity against RCC has been reported [18,19], but studies on the bioactivity of Portuguese propolis in RCC are scarce [19] and lack in the case of propolis-derived fractions, with studies reported to date being only performed on a limited range of cell lines. In the present study, a propolis sample of Portuguese origin was fractionated and its fractions/subfractions assessed for cytotoxicity on different renal cell lines for the first time.

## 2. Results and Discussion

The cytotoxic activity of G18.EE and its fractions was evaluated on three different RCC cell lines-786-O, Caki-2 and A498 (Table 1). The G18.EE-*n*-hexane fraction showed no effect on the viability of any of the cell lines at the tested concentrations. On the other hand, the G18.EE-water fraction exhibited the strongest effect on cell viability, with the lowest IC_50_ values against all cell lines (Table 1). However, this fraction had the lowest selectivity indexes (SI), meaning poor selectivity towards cancer cells. G18.EE and the remaining fractions showed lower cytotoxicity for the human non-neoplastic HK2 renal cells (IC_50_ > 30 µg mL^−1^) and Caki-2 cells. The latter was the least sensitive cancer cell line. Considering all the results (Table 1), the G18.EE-EtOAc fraction was selected for further investigation based on its highest activity, with lower though not statistically significant IC_50_ values than G18.EE-*n*-hexane and G18.EE-*n*-BuOH fractions against both A498 and 786-O cells (0.162 ± 0.000 and 0.271 ± 0.005 µg mL^−1^, respectively) but with higher selectivity indexes.

Propolis has been extensively investigated for its anticancer potential [5,11]. Propolis from Poland and Egypt have been investigated on LNCaP and PC3 prostate cancer cells, respectively, and both samples showed cytotoxicity in a concentration-dependent manner against both cell lines [20,21]. Ethanol extracts of propolis from China and Brazil showed a dose-dependent reduction of the viability of several colon cancer cell lines (CaCo2, HCT116, HT29 and SW480). The IC_50_ values obtained ranged from 4.4 to 41 µg mL^−1^, CaCo2 cells being the least susceptible to both extracts (IC_50_ > 50 µg mL^−1^) [13]. Other studies revealed the potential of Chinese and Japanese propolis on melanoma (A375) and lung (A549) cancer cells (IC_50_ values of 112 and 12 µg mL^−1^, respectively) [22,23]. Methanol and water extracts of propolis from Thailand were tested against the SW620 colorectal cancer cell line, the latter extract being the most active, with a 77% of cell viability reduction when tested at 150 µg mL^−1^ [24]. Altogether, these studies provide strong evidence for the anticancer potential of propolis crude extracts against a wide range of different cancer types.

Propolis extracts from other Portuguese regions (Pereiro, Bragança, Mirandela, Coruche, Aljezur, S. Miguel Island from Azores, and Funchal from Madeira Island) have been tested on different cancer cell lines, including breast (MDA-MB-231, MDA-MB-468 and MCF-7), prostate (DU145 and 22RV1) and glioblastoma (SW1088), with IC_50_ values ranging from 9 to 182 µg mL^−1^ [14,16,17]. A previous study described the effect of methanol extracts of propolis from Bornes and from Fundão (Portugal) on A498 RCC cells (IC_50_ values of 70.8 ± 10.7 and > 100 µg mL^−1^, respectively) [19]. In the present work, we report the activity of a Portuguese propolis sample with remarkable cytotoxicity against tumoral cell lines. The IC_50_ values obtained are 400 times lower than the values found in the literature.

Different fractions (*n*-hexane, chloroform and ethanol residual) obtained from an ethanol extract of Portuguese propolis from Angra do Heroísmo showed IC_50_ values ranging from 5 to 26 µg mL^−1^ on colorectal cancer HCT-15 cells [14]. The *n*-hexane fraction of an EE of propolis from Thailand showed high anticancer activity against different cancer cell lines (SW620, BT474, Hep-G2, Chago and Kato-III) [25], unlike the *n*-hexane fraction used in the previous study, which showed no cytotoxic effect.

The molecular mechanism of the anticancer activity of propolis has yet to be fully understood. Propolis has been reported to decrease cell proliferation and migration, down-regulate NF-kB p65 levels, and increase apoptosis by interfering with the caspase pathway in breast cancer cells [10,11,16]; interfere with the protein expression of cyclins (D1, B1) and cyclin-dependent kinase (CDK) p21, inhibiting proliferation in prostate cancer cells [12]; increase cellular mRNA levels of p21 CIPI and p53 and induce apoptosis in colon cancer cells [13]; disrupt tumor glycolytic metabolism, inhibiting growth and leading to cell death in melanoma and colorectal cancer cells [14,15], among many others [5]. The exact mechanism of action of propolis against RCC cells is still unknown. Only one study, using endothelial cells and RCC4 cells revealed the inhibitory effect of a red propolis polyphenols (RPP) extract on the vascular endothelial growth factor (VEGF) gene expression through destabilization of the HIF1α protein under hypoxic conditions resulting in reduction of angiogenesis. The authors attributed this destabilization to the ability of RPP to diminish the expression of the Cdc42 protein and consequently increase the expression of the pVHL E3 ubiquitin ligase [18].

**Figure 1:**
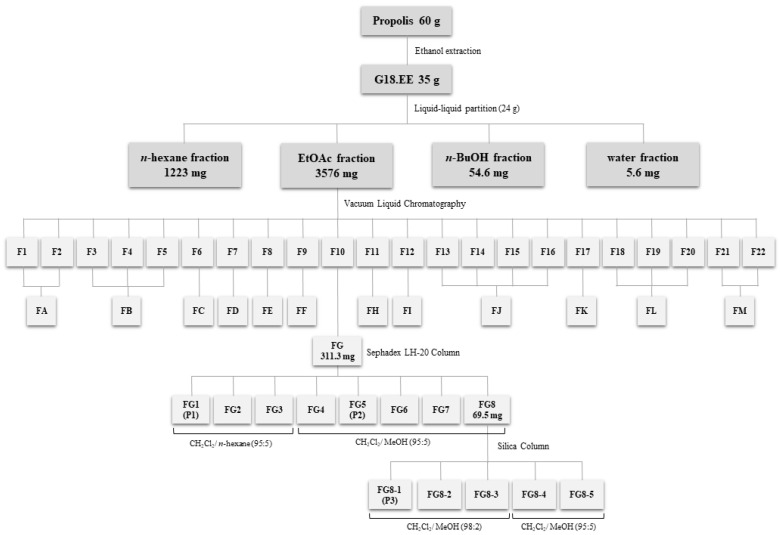
Fractionation of G18.EE and methodologies used for isolation of subfractions and compounds from the G18.EE-EtOAc fraction of Portuguese propolis. G18.EE–ethanol extract of propolis from Gerês collected in 2018; EtOAc–ethyl acetate; BuOH–butanol; F–fraction.

**Figure 2:**
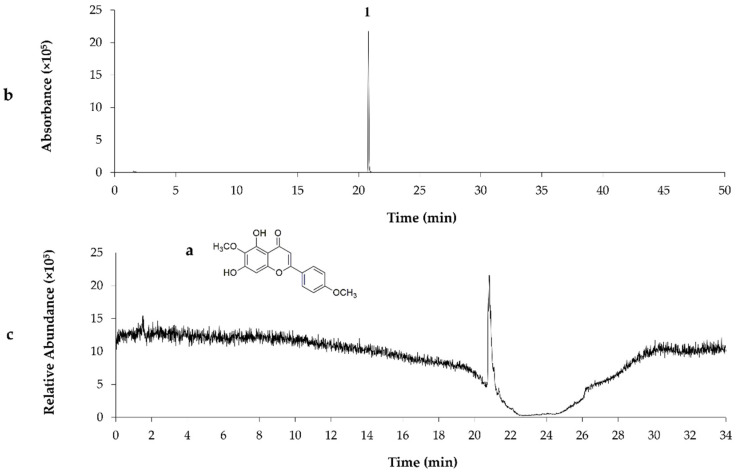
The chemical structure of pectolinarigenin (**a**); LC-MC chromatogram of **P1** isolated from G18.EE-EtOAc fraction using ESI (**b**); TIC chromatogram in negative ion mode (**c**); Pectolinarigenin (**1**).

**Figure 3:**
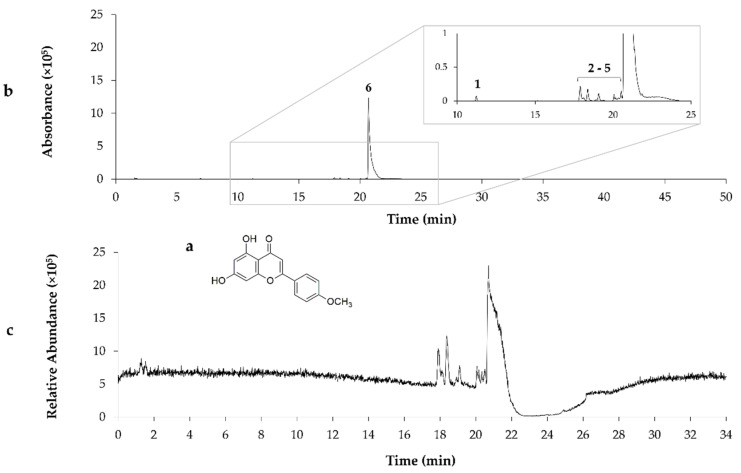
The chemical structure of acacetin (**a**); LC-MC chromatogram of **P2** isolated from G18.EE-EtOAc fraction using ESI (**b**); TIC chromatogram in negative ion mode (**c**); *p*-Coumaric acid (**1**); unknown (**2**); Pinobanksin (**3**); Isorhamnetin (**4**); Quercetin-dimethyl ether (**5**); Acacetin (**6**).

**Figure 4:**
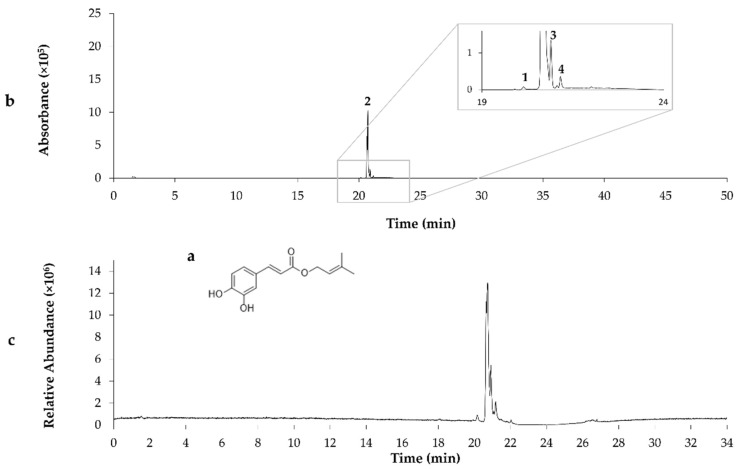
The chemical structure of caffeic acid isoprenyl ester (**a**); LC-MC chromatogram of **P3** isolated from G18.EE-EtOAc fraction using ESI (**b**); TIC chromatogram in negative ion mode (**c**); Quercetin-dimethyl ether (**1**); Caffeic acid isoprenyl ester (**2**); Caffeic acid phenylethyl ester (**3**); Caffeic acid cinnamyl ester (**4**).

Chemical composition of the G18.EE and its fractions was already investigated and published by our research group [26], revealing the presence of compounds such as apigenin, pinobanksin, pinobanksin 3-O-acetate, chrysin, acacetin, CAIE, and pinocembrin. Partitioning of G18.EE-EtOAc fraction led to the isolation of the compound **P1** and eleven subfractions (FG2–FG7 and FG8-1–FG8-5) composed of a mixture of compounds (Figure 1). FG1 (**P1**), FG5 (**P2**) and FG8-1 (**P3**) were selected for further chemical investigation, since they appear as a single spot on the TLC, suggesting the presence of a single compound. **P1** was identified as pectolinarigenin, following NMR (as described in Section 3), MS analysis, and by comparison with previously published data (Table 2; Figure 2) [27]. The compounds present in **P2** and **P3** were identified by comparison of the retention times and values of ESI-MS/MS published in the literature [9], with acacetin and caffeic acid isoprenyl ester as the major compounds in **P2** and **P3**, respectively (Table 2; Figure 3 and Figure 4).

The effect of the subfractions obtained from the G18.EE-EtOAc fraction on the viability of 786-O and A498 cell lines is presented in Table 3. **P1** and **P3** similarly affected the viability of both cell lines, with **P3** being more selective towards both cancer cell lines. **P2** had no effect on the viability of any of the cell lines at the tested concentrations, and 786-O was the most susceptible RCC cell line to both **P1** (IC_50_ = 3.8 ± 0.2 µg mL^−1^; 12.1 ± 0.6 µM) and **P3** (IC_50_ = 3.1 ± 0.01 µg mL^−1^). All subfractions (**P1**–**P3**) showed lower cytotoxicity against 786-O and A498 cell lines when compared to their fraction of origin (G18.EE-EtOAc; Table 1), which is in line with previous observations that the biological properties of propolis can be attributed to not only certain compounds but also to a synergistic effect that may occur between compounds [28].

Compounds present in **P1**, **P2** and **P3** have previously been reported in other propolis samples [9,28,29,30]. Several compounds naturally present in European propolis [29], such as pectolinarigenin, acacetin, caffeic acid phenethyl ester (CAPE), chrysin, galangin, quercetin, apigenin, acacetin, pinobanksin and kaempferol, have demonstrated anticancer activity [5,31,32,33,34,35,36].

Pectolinarigenin was shown to be active against different cancer cell lines [37,38,39,40,41]. To date and to our knowledge, only one study described its anticancer potential on RCC cells (ACHN) with an IC_50_ value of 15.2 µM [42]. The effects of pectolinarigenin on the viability of SK-HEP-1, SMMC-7721 and PLC5 liver cancer cells were also reported (IC_50_ values of 10, 11.59 and 11.97 µM, respectively) [39,40,41] as well as on A549 and Calu-3 lung cancer cells (IC_50_ values of 21.49 and 22.63 µM, respectively) [40], SW620 colon cancer cells (IC_50_ = 13.05 µM) and KATO-III, AGS and MKN28 gastric cancer cells (IC_50_ of 24.31, 124.79 and 96.88 µM, respectively) [37,38].

Acacetin has demonstrated activity on breast cancer - MDA-MB-231 and MCF-7 - (IC_50_ values of 82.75 and 103.91 µM, respectively) [43], liver (SMMC-7721; IC_50_ > 200 µM), lung (A549; IC_50_ = 157.40 µM) and prostate (DU145; IC_50_ = 25 µM) cancer cell lines [44]. Although acacetin appears to be the lead compound of **P2**, no cytotoxic effect was found against any of the RCC at the tested concentrations in the present study (Table 3). The IC_50_ values found for acacetin on other cancer cell lines are in general substantially higher than the maximum concentration of **P2** tested herein and **P2** activity cannot be attributed to the activity of acacetin as a single compound, but to the interaction of several compounds present in the subfraction.

To the best of our knowledge, the present work also shows, for the first time, evidence of the anticancer potential of CAIE, identified as the main compound in **P3**. Further studies on the anticancer activity of this pure compound are warranted.

To explore the cytotoxic activity of the combination of different components of the propolis fractions, two different mixtures were prepared (**P1** + **P2** + **P3** and **P1** + **P3**) with equal parts of each subfraction and tested against the 786-O cell line (Table 4). The **P1** + **P3** combination (IC_50_ = 2.5 ± 0.03 µg mL^−1^) showed no significant differences when compared to each individual subfractions (*p* > 0.05), contrary to what was observed for the **P1** + **P2** + **P3** combination (IC_50_ = 8.6 ± 0.06 µg mL^−1^; *p* ≤ 0.001), with **P1** + **P3** combination showing higher selectivity towards the 786-O cancer cell line. This fact may be explained by the presence of **P2**, for which no activity was detected (IC_50_ > 30 µg mL^−1^). Nevertheless, the effect of these subfractions, individually or mixed, did not surpass the effect shown by the original fraction (EtOAc fraction; Table 1), which is chemically more complex, suggesting that other constituents of this fraction are acting synergistically.

Interestingly, some combinations of propolis-derived compounds have already been studied for their anticancer potential and additive/synergistic effects have been observed [45]. This prompted us to investigate the effect of combining the subfractions isolated in this study on the viability of 786-O cells. The results (Table 4) reflect the complexity of propolis mixtures which can contain cytotoxic compounds and others without anticancer potential, being antagonistic or just diluting the overall cytotoxic effect of the mixture.

## 3. Materials and Methods

### 3.1. Chemicals and Reagents

Analytical grade ethanol, *n*-hexane, ethyl acetate (EtOAc), *n*-butanol (*n*-BuOH) and methanol (MeOH) were obtained from Fisher Scientific, UK. Pre-coated silica gel 60 F_254_ TLC plates (Merck^®^, Darmstadt, Germany) were used for the Thin-layer Chromatography (TLC) analysis. Sephadex^®^ LH 20 was purchased from Sigma–Aldrich (St. Louis, MO, USA). Silica gel 60 (0.063–0.200 mm; Merck^®^, Darmstadt, Germany) was used for column chromatography. Silica gel 60 H (Merck^®^, Darmstadt, Germany) was used for Vacuum Liquid Chromatography (VLC). Dimethyl sulfoxide (DMSO) and DMSO-*d*6 were obtained from Sigma-Aldrich, Portugal.

### 3.2. Propolis Sample

The propolis sample used in this work (coded as G18) was collected in 2018 from an apiary located near the Cávado River, between the villages of Paradela and Sirvozelo, in Montalegre, Gerês, Portugal (41°45′41.62″ N; 7°58′03.34″ W).

### 3.3. Extraction and Fractionation of Propolis

Propolis G18 was extracted, as previously described [9], and a dried ethanol extract (EE) of propolis was obtained (70% yield) after solvent evaporation. G18.EE was stored at 4 °C, in the dark, until further use. G18.EE (4 g) was dissolved in ethanol (20 mL) and deionized water (200 mL) and successively partitioned with *n*-hexane, EtOAc and *n*-BuOH (3 × 400 mL each). Organic layers were pooled, dried over sodium anhydrous sulphate, and concentrated under reduced pressure at 40 °C to obtain the G18.EE-*n*-hexane (1.22 g), G18.EE-EtOAc (3.58 g), G18.EE-*n*-BuOH (54.6 mg) and G18.EE-water (5.6 mg) fractions. The partition process was repeated 6 times, with 4 g of G18.EE each time, in order to obtain satisfactory amounts of fractions for further isolation work. All fractions were stored at 4 °C in the dark until further use and after evaporation of the respective solvent.

#### Isolation of Compounds from the G18.EE-EtOAc Fraction

The total G18.EE-EtOAc fraction obtained (3.58 g) was subjected to VLC, eluting successively with *n*-hexane, *n*-hexane/EtOAc mixtures of increasing polarity, and finally with mixtures of EtOAc and MeOH (up to 50% MeOH in EtOAc). A total of 22 subfractions (F1–F22; Figure 1) were obtained and evaporated to dryness under reduced pressure at 40 °C. Subfractions were pooled according to similar TLC profiles into 13 main subfractions (FA–FM; Figure 1). The subfraction with the highest yield, FG (311.3 mg), eluted with *n*-hexane/EtOAc (1:1), was further fractionated using Sephadex LH 20. Elution started with a relatively non-polar solvent system and then the polarity was increased gradually [CH_2_Cl_2_/*n*-hexane (95:5); CH_2_Cl_2_ (100); CH_2_Cl_2_/MeOH (95:5); CH_2_Cl_2_/MeOH (90:10)]. From this subfraction (FG), eight FG-derived subfractions were obtained (FG1–FG8). FG8 was selected for additional fractionation using silica column with the same solvent system [CH_2_Cl_2_/MeOH (95:5); CH_2_Cl_2_/MeOH (98:2)], resulting in five FG8-derived subfractions (FG8-1–FG8-5). Based on the TLC profiles, three FG-derived subfractions (FG1, FG5 and FG8-1) displayed a single spot each on TLC plate, suggesting the presence of a single compound and thus being selected for the following experiments. Solvents used in the columns for extraction and the respective yields were: FG1 [**P1**; CH_2_Cl_2_/*n*-hexane (95:5); 2.2 mg], FG5 [**P2**; CH_2_Cl_2_/MeOH (95:5); 2.7 mg] and FG8-1 [**P3**; CH_2_Cl_2_/MeOH (98:2); 4.3 mg] (Figure 1).

### 3.4. Chemical Analysis of the Subfractions

#### 3.4.1. UPLC-DAD-ESI/MS^n^ Analysis

Subfractions **P1**, **P2**, and **P3** were analyzed by UPLC-DAD-ESI/MS^n^ following a previously described method [46]. The dried subfractions were dissolved in methanol to a final concentration of 10 mg mL^−1^ and 2 µL were used for injection. The Ultimate 3000 apparatus was equipped with an Ultimate 3000 Diode Array Detector (Dionex Co., Sunnyvale, CA, USA) and coupled to an ion trap mass spectrometer LTQ XL (Thermo Scientific, Waltham, MA, USA) equipped with an ESI source. Analysis was run on a Hypersil Gold (Thermo Scientific, Waltham, MA, USA) C18 column (100 mm length; 2.1 mm i.d.; 1.9 μm particle diameter, end-capped) at 30 °C. The mobile phase was composed of (A) water/formic acid (99:1; *v*/*v*) and (B) acetonitrile/formic acid (99:1; *v*/*v*). The solvent gradient started with 5–40% B (0–15 min), followed by 40–100% B (15–19 min), and returning to the initial conditions. The flow rate was 200 μL min^–1^ and UV–Vis spectral data for all peaks were accumulated in the range 200–500 nm. The nitrogen (>99% purity) gas pressure used for mass spectrometry analysis was 520 kPa (75 psi). The mass spectrometer was operated in negative-ion mode with ESI needle voltage set at 5.00 kV and an ESI capillary temperature of 275 °C. The full scan covered the mass range from *m*/*z* 100 to 2000. CID–MS/MS and MS^n^ experiments were simultaneously acquired for precursor ions using helium as the collision gas with collision energy of 25–35 arbitrary units. Control and data acquisition were carried out with the Thermo Xcalibur Qual Browser data system (Thermo Scientific, Waltham, MA, USA). Compounds in the subfractions (**P1**–**P3**) were identified based on a literature comparison of the ESI-MS/MS data [9].

#### 3.4.2. NMR Analysis

NMR spectra of **P1** were recorded in a Bruker Advance II 400 spectrometer, at 400 MHz (^1^H) and 100 MHz (^13^C), at 20 °C (see Supplementary Figures S1 and S2). The chemical shifts (*δ*) were recorded in parts per million (ppm) relative to the residual solvent signal (DMSO-*d*6).

5,7-dihydroxy-6-methoxy-2-(4-methoxyphenyl)-4H-chromen-4-one (pectolinarigenin; **P1**). Yellow amorphous solid. ^1^H NMR (DMSO-*d*6, 400 MHz): *δ* = 3.77 (3H, s, OCH_3_), 3.85 (3H, s, OCH_3_), 6.61 (1H, s), 6.86 (1H, s), 7.10 (2H, d, *J* = 7.2 Hz), 8.03 (2H, d, *J* = 6.8 Hz), 10.72 (1H, s), 13.02 (1H, s). ^13^C NMR (DMSO-*d*6, 100 MHz): *δ* = 55.5, 59.9, 94.3, 103.0, 104.1, 114.6 (2C), 122.8, 128.3 (2C), 131.4, 152.4, 152.7, 157.4, 162.3, 163.3, 182.1 [27].

### 3.5. Cell Lines, Media and Growth Conditions

Four human renal cell lines-HK2 (renal non-neoplastic cells) and 786-O, Caki-2 and A498 (RCC cells) were obtained from the American Type Culture Collection (ATCC). The HK2 and 786-O cell lines were cultured in RPMI 2.0 g/ L NaHCO_3_ without L-glutamine (RPMI 1640, Biochrom GmbH, Berlin, Germany). The Caki-2 cell line was cultured in McCOY’s high glucose, L-glutamine, bacto-peptone, and phenol red without sodium pyruvate and HEPES (McCOY’s 5A, Biochrom GmbH). The A498 was cultured in MEM with 20 mM HEPES without NaHCO_3_ and L-glutamine (MEM Earle’s, Biochrom GmbH). In addition, 1% of an antibiotic preparation (penicillin-streptomycin, Gibco) and 10% heating activated fetal bovine serum (FBS Superior, Biochrom GmbH) were used as supplements for the culture media. Cultures were grown at 37 °C and 5% CO_2_ in a humidified incubator.

To prepare cells for the cytotoxicity assay, flasks with sub-confluent cells were washed with phosphate-buffer 1× (PBS Dulbecco, Merck^®^) and then were detached from the flask with trypsin (TrypLE™ Express, Gibco, New York, USA) at 37 °C. Medium was then added to the flask for trypsin inactivation. Cells were collected into a 15 mL Falcon tube and centrifuged for 5 min at 900 rpm, at 4 °C. Fresh medium was used to resuspend the cells, and 10 µL of the cell suspension were collected for subsequent addition of 20 µL of trypan blue (Trypan Blue Solution 0.4%, Gibco) for cell counting (Neubauer chamber).

### 3.6. Cytotoxicity Assay and Selectivity Index (SI) Calculation

Propolis cytotoxicity was assessed by the sulforhodamine B (SRB) assay as previously described [47], which estimates cell biomass based on cell protein content. Briefly, each cell line was plated at an appropriate density (2000 cells/well for A498 and 786-O or 3000 cells/well for Caki-2 and HK2) in 96-well plates for 24 h. The dried extract and subfractions (by solvent evaporation) were dissolved in DMSO for the cells’ treatments. Cells were then treated for 72 h with different concentrations of G18.EE (0.005–100 µg mL^−1^), each of the four fractions (*n*-hexane, EtOAc, *n*-BuOH and water fractions; 0.005–30 µg mL^−1^), each of the three subfractions (**P1–P3**; 0.005–30 µg mL^−1^) and two combinations of the subfractions with equal parts of each subfraction (**P1** + **P2** + **P3** and **P1** + **P3**; 0.005–30 µg mL^−1^). Controls were performed with 0.25% of DMSO (vehicle) for IC_50_ determination (concentration that inhibits cell viability in 50%). Following this incubation period, the adherent cells were fixed by adding 50 µL of cold trichloroacetic acid 10% (TCA, PanReac, Barcelona, Spain) and incubating for 60 min, at 4 °C. The plates were then washed with deionized water, dried overnight, and 50 µL of 0.1% SRB solution (Sigma–Aldrich) in 1% acetic acid (PanReac) were added to each plate well. After 30 min incubation at room temperature, unbound SRB was removed by washing with 1% acetic acid and air-dried overnight. Bound SRB was solubilized with 100 µL of 10 mM Tris base (Trizma base, Merck^®^) and the absorbance was measured at 490 nm in a microplate reader (Tecan Infinite M200, Grödig/Salzburg, Austria). IC_50_ values were determined by transforming the absorbance values into a sigmoidal dose-response curve and extracting the concentration of the extracts/fractions needed to inhibit the viability of the kidney cells by 50%, using the GraphPad Prism Software Version 6.0. The selectivity index (SI) was calculated based on the IC_50_ values obtained for the samples against the cell lines, using the following formula:

SI = (IC_50_ HK2 cell line − IC_50_ cancer cell line)/IC_50_ cancer cell line

Cytotoxicity is higher for the cancer cell line than for the non-neoplastic HK2 cell line when SI values > 1.

### 3.7. Statistical Analysis

Experiments were performed in triplicate and repeated at least three times for IC_50_ determination using the GraphPad Prism 5 software for logarithmic transformation after applying a sigmoidal dose-response non-linear regression. For multiple comparisons one-way ANOVA supplemented with post-hoc Tukey test was performed. Different letters mean statistical differences (*p* < 0.05) between mean values.

## 4. Conclusions

Cancer is one of the leading causes of death worldwide and is expected to cause more than 16 million deaths in 2040 [1]. Traditional cancer treatments include surgery, radiation, and chemotherapy, which are associated with significant side effects [2]. Comparisons with the existent literature are difficult to make considering the lack of studies on RCC cell lines, the different methodologies adopted for cytotoxicity assessment, and the variety of treatment conditions implemented by few published studies. Nevertheless, the propolis samples studied in this work show a strong anticancer effect on different RCC cells, presenting very low IC_50_ values, and importantly, very high selectivity indexes, when compared to the data available in the literature. This suggests that Portuguese propolis from Gerês, as well as its chemical constituents, could be used as alternatives or adjuvants to conventional anticancer treatments. They could be also used to help tackle drug-resistance and toxic effects by reducing the dose of the therapeutic drugs commonly used in RCC treatment.

## Figures and Tables

**Table 1:** IC_50_ values and respective selectivity indexes of G18.EE and their fractions against RCC cells (72 h treatment).

Propolis Fractions	IC_50_ ± SD (µg mL^−1^) *				SI ^α^ (vs. HK2)		
	Caki-2	A498	786-O	HK2 (Non-Neoplastic)	Caki-2	A498	786-O
***n*** **-hexane**	>30	>30	>30	>30	0	0	0
**EtOAc**	>30	0.162 ± 0.000 ^c,d^	0.271 ± 0.005 ^c,d^	>30	0	>184.2	>109.7
***n*** **-BuOH**	>30	0.239 ± 0.001 ^c,d^	0.341 ± 0.003 ^c,d^	>30	0	>124.5	>87.0
**water**	0.573 ± 0.030 ^c^	0.085 ± 0.001 ^d^	0.199 ± 0.013 ^c,d^	0.229 ± 0.000 ^c,d^	−0.6	1.7	0.2
**G18.EE (propolis extract)**	0.765 ± 0.041 ^c^	0.153 ± 0.004 ^c,d^	87.170 ± 0.707 ^a^	49.185 ± 0.106 ^b^	63.3	320.5	−0.4

^α^ SI (Selectivity Index) = (IC_50_ HK2 cell line-IC_50_ cancer cell line)/IC_50_ cancer cell line; * Experiments were done in triplicate and repeated at least three times. Results are expressed as mean of IC_50_ values ± standard deviation (SD). One-way ANOVA followed by Tukey test was performed to assess significance. Mean values sharing the same superscript letters (^a^, ^b^, ^c^ or ^d^) have no statistically significant differences.

**Table 2:** The detection of compounds present in **P1**, **P2** and **P3**, isolated from G18.EE-EtOAc, following LC-MS analysis.

Subfractions	Compound Code	t_R_ (min)	λmax (nm)	[M-H]^−^ m/z	**Main Fragments**	Compound Detected
**P1**	**1**	20.8	275, 330	313	-	Pectolinarigenin
**P2**	**1**	11.2	309	163	119, 145, 108	*p*-Coumaric acid
	**2**	17.9	281, 334	299	-	unknown
	**3**	19.1	291	271	253, 225	Pinobanksin
	**4**	20.1	259, 368	315	300	Isorhamnetin
	**5**	20.2	254, 368	329	314	Quercetin-dimethyl ether
	**6**	20.8	268, 329	283	269	Acacetin
**P3**	**1**	20.2	253, 368	329	314	Quercetin-dimethyl ether
	**2**	20.7	298, 325	247	179, 135	Caffeic acid isoprenyl ester
	**3**	20.9	298, 325	283	179, 135	Caffeic acid phenylethyl ester
	**4**	21.2	295, 325	295	178, 134, 251, 211	Caffeic acid cinnamyl ester

**Table 3:** **The** IC_50_ values and respective selectivity indexes of subfractions **P1**, **P2** and **P3** isolated from the G18.EE-EtOAc fraction against RCC cells (72 h treatment).

Subfractions	IC_50_ ± SD (µg mL^−1^/ µM) *			SI ^α^ (vs. HK2)	
	786-O	A498	HK2 (Non-Neoplastic)	786-O	A498
**P1** **(** **pectolinarigenin** **)**	3.8 ± 0.2/ 12.1 ± 0.6 ^e^	11.8 ± 0.04/ 37.8 ± 0.1 ^d^	13.2 ± 0.09/ 42.2 ± 0.3 ^c,d^	2.5	0.1
**P2**	>30	>16	29.7 ± 0.3 ^a^	<0	<0.9
**P3**	3.1 ± 0.01 ^e^	11.4 ± 0.1 ^d^	24.9 ± 0.4 ^b^	7.1	1.2

^α^ SI (Selectivity Index) = (IC_50_ HK2 cell line-IC_50_ cancer cell line)/ IC_50_ cancer cell line; * Experiments were done in triplicate and repeated at least three times. Results are expressed as mean of IC_50_ values ± standard deviation (SD). One-way ANOVA followed by Tukey test was performed to assess significance. Mean values sharing the same superscript letters (^a^, ^b^, ^c^, ^d^ or ^e^) have no statistically significant differences.

**Table 4:** **The** IC_50_ values and respective selectivity indexes of the combinations of the subfractions (in equal parts) obtained from the G18.EE-EtOAc fraction (72 h treatment) in RCC cells.

Mixtures	IC_50_ ± SD (µg mL^−1^) *		SI ^α^ (vs. HK2)
	786-O	HK2 (Non-Neoplastic)	786-O
**P1 + P2 + P3**	8.6 ± 0.06 ^c^	20.0 ± 0.08 ^a^	1.3
**P1 + P3**	2.5 ± 0.03 ^d^	15.7 ± 0.11 ^b^	5.4

^α^ SI (Selectivity Index) = (IC_50_ HK2 cell line-IC_50_ cancer cell line)/ IC_50_ cancer cell line; * Experiments were done in triplicate and repeated at least three times. Results are expressed as mean of IC_50_ values ± standard deviation (SD). One-way ANOVA followed by Tukey test was performed to assess significance. Mean values sharing the same superscript letters (^a^, ^b^, ^c^ or ^d^) have no statistically significant differences.

## Data Availability

Data is contained within the article.

## Supplementary Materials

The following supporting information can be downloaded at: https://www.mdpi.com/article/10.3390/molecules27134001/s1, Figure S1: ^1^H NMR spectrum (400 MHz) for **P1** (pectolinarigenin) in DMSO-*d*6; Figure S2: ^13^C NMR spectrum (100 MHz) for **P1** (pectolinarigenin) in DMSO-*d*6.
