# Supplementary material for: Selective Cytotoxicity of Portuguese Propolis Ethyl Acetate Fraction towards Renal Cancer Cells
Source: Molecules. 2022 Jun 22;27(13):4001. doi: 10.3390/molecules27134001 (PMC9268251; doi:10.3390/molecules27134001)
Supplement: Supplementary file 1 [file molecules-27-04001-s001.zip › molecules-1768258-supplementary.pdf]

Selective Cytotoxicity of Portuguese Propolis Ethyl Acetate Fraction towards Renal Cancer Cells

Ana Sofia Freitas[1,2,3], Marta Costa[4,5], Olívia Pontes[4,5], Veronique Seidel[6], Fernanda Proença[7], Susana M. Cardoso[8], Rui Oliveira[2,3], Fátima Baltazar[4,5,*], Cristina Almeida-Aguiar[2,3,*]

[1] CITAB, Centre for the Research and Technology of Agro-Environmental and Biological Sciences, Department of Biology, University of Minho, 4710-057 Braga, Portugal; anasofiapfreitas@gmail.com (A.S.F.)

[2] Department of Biology, School of Sciences, University of Minho, Campus de Gualtar, 4710-057 Braga, Portugal

[3] CBMA, Centre of Molecular and Environmental Biology, Department of Biology, University of Minho, 4710-057 Braga, Portugal; ruipso@bio.uminho.pt (R.O.)

[4] Life and Health Sciences Research Institute (ICVS), School of Medicine, University of Minho, Campus of Gualtar, 4710-057 Braga, Portugal; martafcosta@med.uminho.pt (M.C.); oliviaepontes@gmail.com (O.P.)

[5] ICVS/3B's-PT Government Associate Laboratory, 4710-057 Braga/ 806-909 Guimarães, Portugal

[6] Natural Products Research Laboratory, Strathclyde Institute of Pharmacy and Biomedical Sciences, University of Strathclyde, Glasgow G4 0RE, UK; veronique.seidel@strath.ac.uk (V.S.)

[7] Department of Chemistry, University of Minho, Campus of Gualtar, 4710-057 Braga, Portugal; fproenca@quimica.uminho.pt (F.P.)

[8] LAQV-REQUIMTE, Department of Chemistry, University of Aveiro, 3810-193 Aveiro, Portugal; susanacardoso@ua.pt (S.M.C.)

* Correspondence: fbaltazar@med.uminho.pt (F.B.); cristina.aguiar@bio.uminho.pt (C.A.-A.); Tel.: +351-253601513 (C.A.-A.)

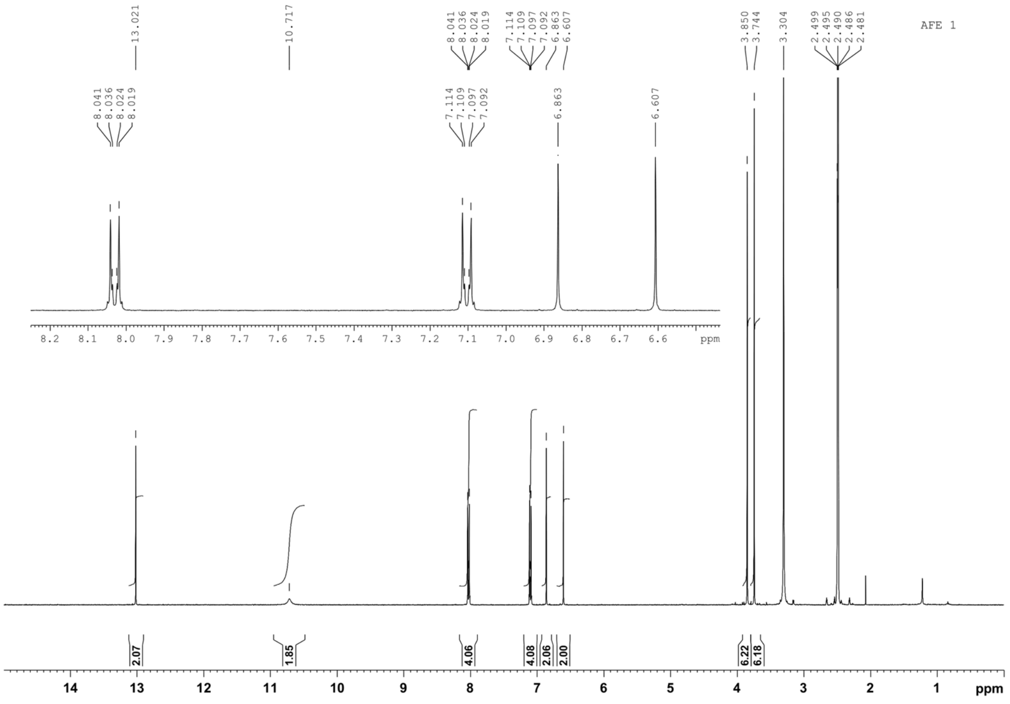

Figure S1. ^{1}H NMR spectrum (400 MHz) for **P1** (pectolinarigenin) in DMSO-*d*6.

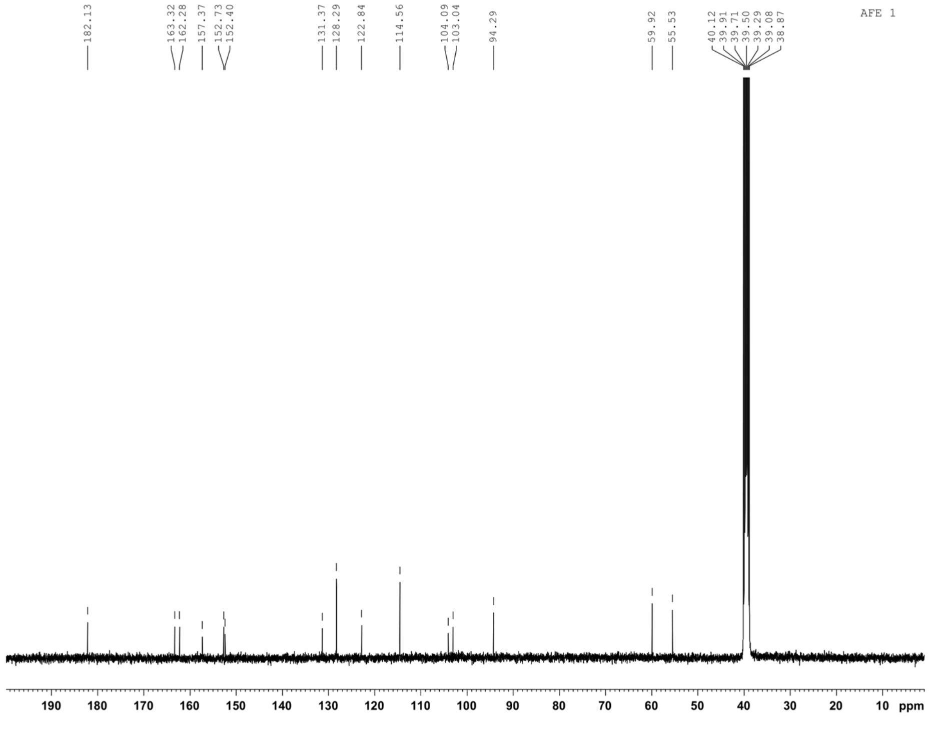

Figure S2. ^{13}C NMR spectrum (100 MHz) for **P1** (pectolinarigenin) in DMSO-*d*6.
